# Long-term effect of neoadjuvant denosumab treatment in high-risk early breast cancer (GeparX)

**DOI:** 10.1016/j.esmoop.2025.105915

**Published:** 2025-11-27

**Authors:** T. Link, M. Reinisch, M. Just, M. Untch, N. Filmann, O. Stötzer, C. Denkert, V. Bjelic-Radisic, P. Wimberger, M. Thill, K. Rhiem, J. Huober, C. Solbach, C. Hanusch, K. Engels, P.A. Fasching, A. Schneeweiss, V. Nekljudova, J. Holtschmidt, J.-U. Blohmer, S. Loibl

**Affiliations:** 1 Department of Gynecology and Obstetrics, Medical Faculty and University Hospital Carl Gustav Carus, und Nationales Centrum für Tumorerkrankungen (NCT), Technische Universität Dresden, Dresden, Germany; 2 Breast Unit, University Hospital Mannheim, Mannheim, Germany; 3 Onkologische Schwerpunktpraxis Bielefeld, Bielefeld, Germany; 4 Helios Klinikum Berlin-Buch, Berlin, Germany; 5 GBG c/o GBG Forschungs GmbH, Neu-Isenburg, Germany; 6 Gemeinschaftspraxis Hämatologie/Intern. Onkologie, München, Germany; 7 Institut für Pathologie, Philipps-University Marburg and University Hospital Marburg (UKGM)—Universitätsklinikum Marburg, Marburg, Germany; 8 Breast Unit, University Hospital Helios, University Witten Herdecke, Wuppertal, Germany; 9 Department of Gynecology and Gynecological Oncology, Agaplesion Markus Krankenhaus, Frankfurt, Germany; 10 Zentrum Familiärer Brust- und Eierstockkrebs, Universitätsklinikum Köln, Köln, Germany; 11 Kantonsspital St. Gallen, St. Gallen, Switzerland; 12 Breast Center, Department of Gynecology and Obstetrics, University Hospital, Goethe-University Frankfurt, Frankfurt, Germany; 13 Rotkreuzklinikum, München, Germany; 14 MVZ Pathologie, Zytologie und Molekularpathologie Neuss, Neuss, Germany; 15 University Hospital Erlangen, Comprehensive Cancer Center Erlangen-EMN, Department of Gynecology and Obstetrics, Erlangen, Germany; 16 National Center for Tumor Diseases, University Hospital and German Cancer Research Center, Heidelberg, Germany; 17 Gynäkologie mit Brustzentrum, Charité-Universitätsmedizin Berlin, Berlin, Germany; 18 Goethe University Frankfurt, Frankfurt am Main, Germany

**Keywords:** breast cancer, denosumab, GeparX trial, neoadjuvant chemotherapy, nab-paclitaxel schedules, survival outcomes

## Abstract

**Background:**

In the GeparX trial (NCT02682693), neoadjuvant denosumab, in addition to either weekly or days 1 and 8 (d1,8) q22 nab-paclitaxel (nPac)-based chemotherapy, did not improve the pathological complete response (pCR) rate in early high-risk breast cancer patients, while the concomitantly applied weekly nPac regimen resulted in a significantly higher pCR rate compared with the interrupted regimen.

**Patients and methods:**

GeparX is a randomized, open-label, phase II study comparing neoadjuvant treatment with or without denosumab and two different nPac schedules. Invasive disease-free survival (iDFS), distant disease-free survival (DDFS), overall survival (OS), and locoregional recurrence-free interval (LRRFI) were considered as time-to-event endpoints.

**Results:**

After a median follow-up of 62.3 months, there was no statistically significant difference in iDFS [hazard ratio (HR) 0.86, 95% confidence interval (CI) 0.62-1.21, *P* = 0.39], DDFS (HR 0.77, 95% CI 0.54-1.11, *P* = 0.16), LRRFI (HR 1.41, 95% CI 0.76-2.63, *P* = 0.28), and OS (HR 0.81, 95% CI 0.50-1.33, *P* = 0.41) between denosumab-treated and non-denosumab-treated patients. However, numerically fewer distant relapses occurred in patients with denosumab treatment (9.2% versus 13.8%). Denosumab treatment resulted in a significant risk reduction of 36% for distant relapse in the multivariate analysis (DDFS; HR 0.64, 95% CI 0.43-0.93, *P* = 0.02). There was no overall differential impact of the two neoadjuvant chemotherapy (NACT) regimens (nPac weekly or nPac d1,8 q22) on long-term outcome in the total study population. Triple-negative breast cancer (TNBC) patients without a pCR (non-pCR) had a significantly worse iDFS (HR 0.22, 95% CI 0.12-0.39, *P* < 0.0001), with a trend toward improved iDFS in those receiving weekly nPac at the 5-year landmark (iDFS rate at 60 months: 58.4% versus 72%).

**Conclusions:**

Denosumab as part of neoadjuvant therapy, although not improving the pCR rate, significantly reduced the risk of distant relapses. Other long-term outcome parameters did not differ between the treatment arms. TNBC patients, especially when not achieving pCR, seem to benefit from weekly nPac.

## Highlights

• GeparX is the first randomized trial to investigate neoadjuvant denosumab in patients with high-risk early breast cancer.
• This preplanned 5-year follow-up analysis assessed iDFS and DDFS, OS, and recurrence patterns.
• Denosumab treatment resulted in a significant reduction in the risk of distant relapse in multivariate analysis.
• These findings suggest a potential long-term anti-metastatic effect of short-course neoadjuvant RANKL inhibition.
• The data support further exploration of denosumab as a component of risk-adapted strategies in early breast cancer.

## Introduction

Over the past two decades, the use of neoadjuvant chemotherapy (NACT) has experienced a notable surge, entailing a transformative shift in therapeutic strategies across multiple cancer types. In the context of breast cancer, NACT was shown to minimize the need for extensive surgical procedures.^1^, ^2^, ^3^ Moreover, NACT not only facilitates an early assessment of treatment response but also aids in guiding subsequent adjuvant interventions.^4^, ^5^, ^6^ Ongoing clinical trials try to further optimize treatment modalities for improving NACT efficacy.^7^ The GeparSepto trial, for instance, showed a notable increase in pathological complete response (pCR) rates with nab-paclitaxel (nPac) compared with solvent-based paclitaxel, albeit accompanied by a higher incidence of peripheral neuropathy (PNP), necessitating a dose reduction from 150 mg/m^2^ to 125 mg/m^2^.^8^ The pCR increase translated into an improved disease-free survival (DFS) especially for patients with triple-negative breast cancer (TNBC) but also hormone receptor-positive and human epidermal growth factor receptor 2 (HER2)-negative breast cancer.^9^

Receptor activator of nuclear factor-kappa B (RANK) ligand (RANKL) is a member of the tumor necrosis factor receptor family and regulates bone homeostasis by promoting the maturation of bone-resorbing osteoclasts.^10^, ^11^, ^12^ As the RANKL/RANK signaling axis is a mediator of progesterone-driven mammary epithelial cell proliferation, there are multiple lines of evidence that this pathway also contributes to the initiation and progression of breast cancer as well as to the formation of bone metastasis.^13^, ^14^, ^15^, ^16^ Denosumab, an inhibitor of RANKL, is approved for the treatment of osteoporosis and prophylaxis of skeletal morbidity associated with metastatic bone disease.^17^ In the adjuvant setting, denosumab was reported to prevent treatment-induced bone loss, to reduce bone metastases, and to improve breast cancer survival in hormone receptor-positive/HER2-negative breast cancer.^18^, ^19^, ^20^ Furthermore, adjuvant denosumab showed a 20%-25% improvement in secondary outcome-related endpoints, with a 3.5 percentage point benefit for DFS at 9 years of follow-up in the ABCSG-18 study.^21^^,^^22^ However, the effect of denosumab in a neoadjuvant setting was unknown.

The GeparX trial (NCT02682693), utilizing a 2 × 2 factorial design, explored for the first time the potential clinical benefits of denosumab in the neoadjuvant setting among high-risk early breast cancer patients, assessing the pCR rate (ypT0 ypN0) as primary endpoint.^23^ In this trial, denosumab was included as an add-on to anthracycline/taxane-based NACT, among two different schedules of nPac administration [weekly versus days 1 and 8 (d1,8) every 3 weeks]. Denosumab did not improve the pCR rate, irrespective of the underlying breast cancer subtype.^23^ In a translational substudy of GeparX, we consistently found that RANK expression status did not allow differentiation between breast cancer subtypes in terms of their response to denosumab. Nevertheless, we identified RANK expression as an independent predictor of NACT effectiveness, particularly in patients with a luminal-like subtype.^24^ As a further key finding of the GeparX trial, weekly nPac regimen resulted in a significantly higher pCR rate (44.9% versus 39.0%) in the total study population and particularly in TNBC patients (60.4% versus 50.0%), albeit with a concomitant increase in non-hematologic toxicity.^23^

In the present analysis, we investigated the effects of denosumab neoadjuvant treatment and the two different nPac regimens on the patient’s 5-year long-term outcome, using the following time-to-event (TTE) endpoints: (i) invasive disease-free survival (iDFS), (ii) distant disease-free survival (DDFS), (iii) locoregional recurrence-free interval (LRRFI), and (iv) overall survival (OS).

## Patients and methods

### Study participants and clinical setting

GeparX (NCT02682693), a phase IIb study, was conducted by the German Breast Group (GBG) in partnership with the Breast Study Group of the ‘Arbeitsgemeinschaft für Gynäkologische Onkologie’ (AGO-B) across 38 sites in Germany. The study protocol had been approved by the responsible ethics committees and health authority. This study conformed to the ethical principles for clinical research involving human participants outlined in the Declaration of Helsinki. All participants provided written informed consent at enrollment. Baseline patient and tumor characteristics as well as major inclusion criteria have been reported previously.^23^

### Study design

Estrogen receptor (ER), progesterone receptor, and HER2 status were centrally assessed. The trial design has previously been reported in detail.^23^ Briefly, patients were first randomly assigned using Pocock minimization to neoadjuvant therapy with or without the addition of denosumab. Subsequently, patients underwent a second randomization, also using Pocock minimization, where the first randomization outcome (denosumab) served as an additional minimization factor for the chemotherapy regimen. In all study arms, treatment continued until surgery, disease progression, unacceptable toxicity, patient withdrawal of consent, or termination by the sponsor.

### Treatments

Denosumab 120 mg was administered subcutaneously every 4 weeks for six cycles. nPac 125 mg/m^2^ was administered weekly for 12 weeks or on d1,8 every 3 weeks for four cycles (8 doses in 12 weeks), both followed by epirubicin/cyclophosphamide (EC) at 90/600 mg/m^2^ every 2 or 3 weeks, based on investigator’s discretion. HER2-positive disease received the trastuzumab biosimilar ABP 980 6 mg/kg (loading dose, 8 mg/kg) and pertuzumab 420 mg (loading dose, 840 mg), every 3 weeks for eight cycles, simultaneously with chemotherapy for at least four applications (according to label). Patients with TNBC received weekly carboplatin with an area under the curve of 2 concurrently with both nPac regimens.

### Endpoint and statistical analysis

TTE endpoints—iDFS, DDFS, OS, and LRRFI—were defined as the period between randomization and the first event, according to Hudis et al.^25^ These endpoints were analyzed at the study’s conclusion using data from the GBG patient follow-up registry. TTE endpoints were evaluated in the intention-to-treat set for all treatment arms and stratified subpopulations. Survival probabilities were estimated using the Kaplan–Meier method, with two-sided 95% confidence intervals (CIs). Stratified log-rank tests were used to compare TTE endpoints between treatments arms. Hazard ratios (HRs) were determined using univariate and multivariate Cox regression models.

Multivariable Cox regression models including pCR as a covariate were prespecified as landmark analyses, with surgery as the time origin, to account for pCR assessment at that time and to estimate prognosis conditional on response. Models were adjusted for tumor size, nodal status, grade, clinical stage, chemotherapy schedule, subtype (hormone receptor positive/HER2 negative, TNBC, HER2 positive), and pCR. This approach served as the primary modeling strategy for iDFS, DDFS, and OS. As a sensitivity analysis, we also carried out baseline-only Cox models using randomization as the time origin and covariates limited to pre-randomization factors, excluding pCR. These complementary models are briefly reported in the Results section and detailed in the Supplementary Material, available at https://doi.org/10.1016/j.esmoop.2025.105915. For LRRFI and *post hoc* analyses of bone metastasis incidence, cumulative incidence functions with Gray’s test and the Fine–Gray method were used. No adjustments for multiple comparisons were made in subgroup analyses. The significance threshold was set at two-sided alpha of 0.05.

## Results

### Baseline demographics and patient characteristics

**Figure 1:**
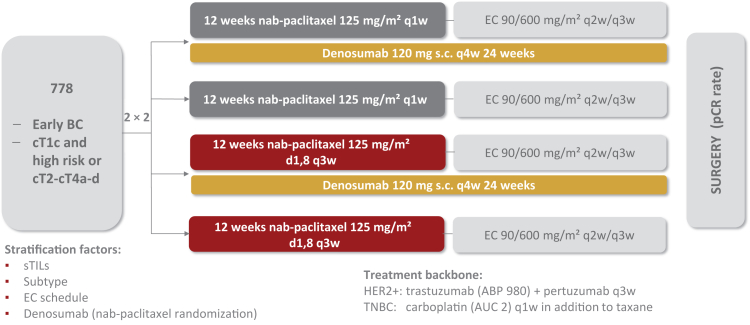
**Trial design of GeparX.** Patients were eligible for inclusion with cT2-cT4a-d or cT1c and cN positive or cT1c and pathological positive nodal status by sentinel lymph node positive or cT1c and ER/PgR negative or cT1c and Ki67 > 20% or cT1c and HER2 positive. BC, breast cancer; c, clinical; d1,8, days 1 and 8; EC, epirubicin and cyclophosphamide; ER, estrogen receptor; HER2, human epidermal growth factor receptor 2; N, nodal; PgR, progesterone receptor; pCR, pathological complete response; s.c., subcutaneously; SLN, sentinel lymph node; T, tumor; sTIL, tumor-infiltrating lymphocyte; TNBC, triple-negative breast cancer.

Between February 2017 and March 2019, a total of *n* = 780 patients were randomly assigned to receive either denosumab or no denosumab (first randomization step) and in a second randomization to receive either nPac weekly or nPac d1,8 q22 (Figure 1). Baseline characteristics were well balanced between the treatment arms, excluding any patient selection bias. The distribution of breast cancer subtypes was as follows: 310/780 patients (39.7%) had hormone receptor-positive, HER2-negative disease, 317/780 (40.6%) had TNBC, and 153/780 (19.6%) had HER2-positive disease (as previously reported^23^). The median follow-up at data cut-off was 62.3 months (interquartile range 51.9-68.3 months).

### Long-term effect on the patient’s outcome

**Figure 2:**
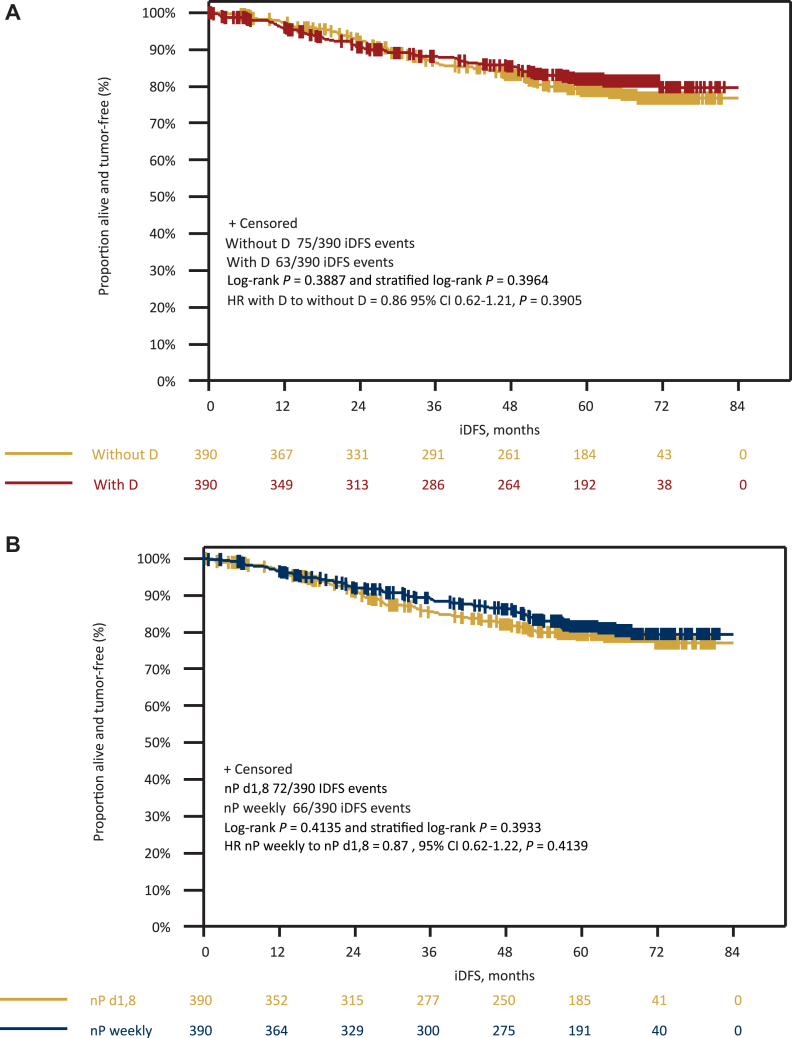
**Invasive disease-free survival.** Kaplan–Meier curves for iDFS according to (A) denosumab randomization and (B) nPac randomization. CI, confidence interval; D, denosumab; d1,8, days 1 and 8; HR, hazard ratio; iDFS, invasive disease-free survival; nP, nab-paclitaxel.

**Figure 3:**
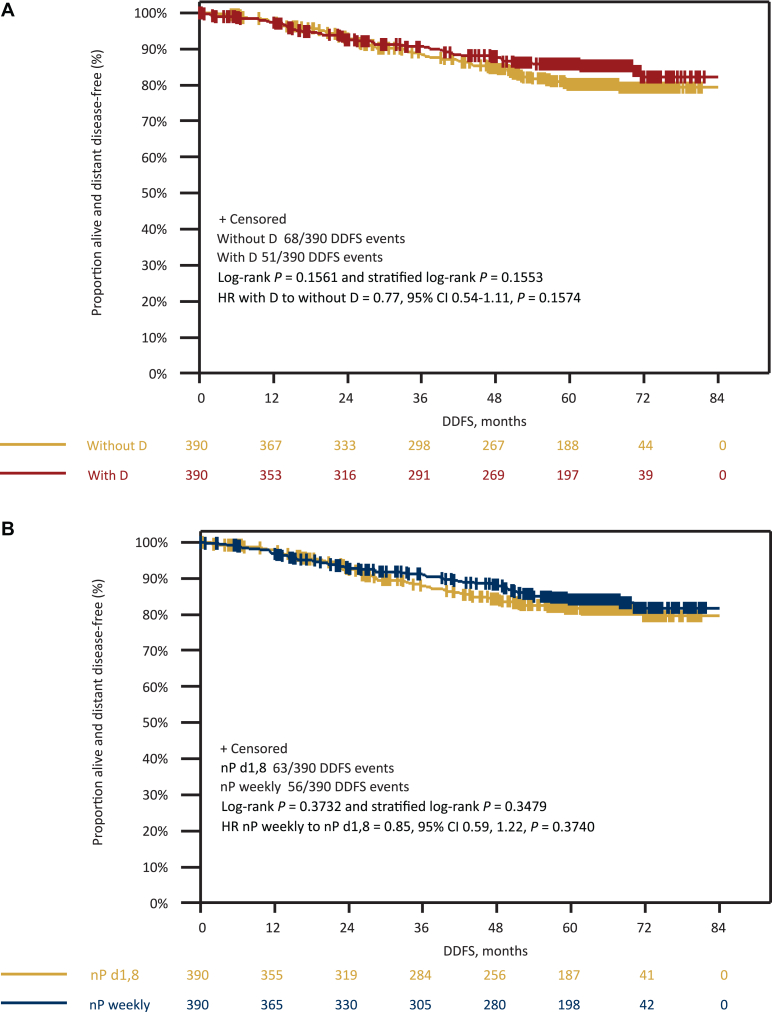

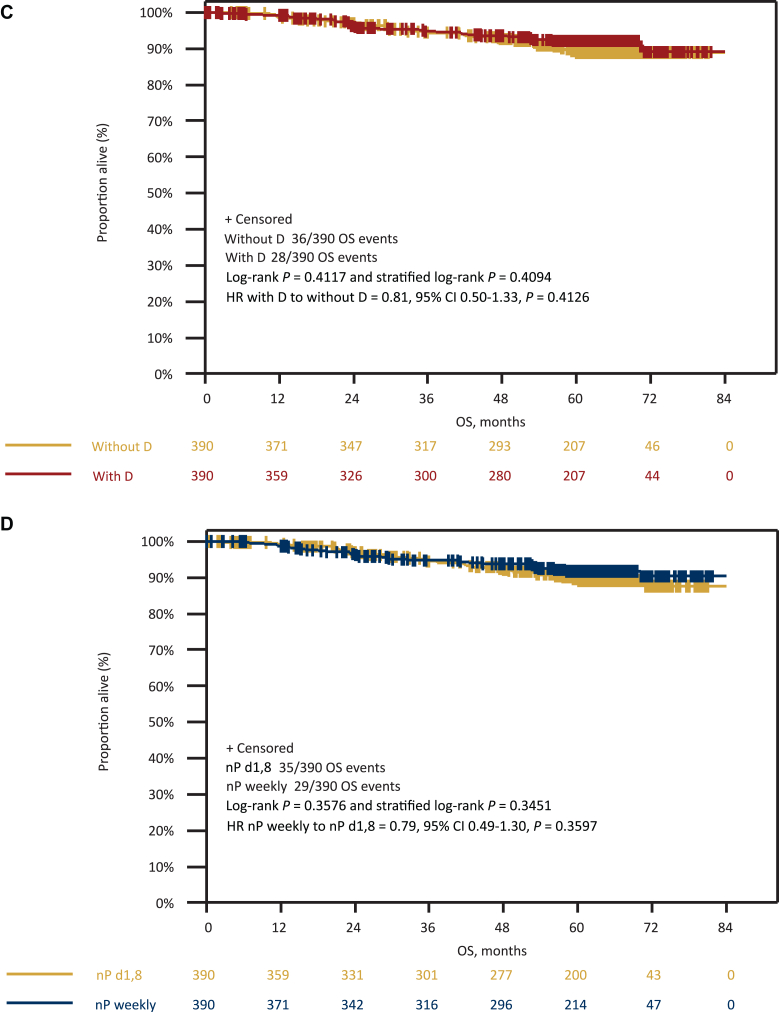
**Distant disease-free survival and overall survival.** Kaplan–Meier curves for DDFS according to (A) denosumab and (B) nPac randomization; OS according to (C) denosumab and (D) nPac randomization. CI, confidence interval; D, denosumab; d1,8, days 1 and 8; DDFS, distant disease-free survival; HR, hazard ratio; nP, nab-paclitaxel; OS, overall survival.

Overall, 138 iDFS events (63 with denosumab and 75 without denosumab; 66 with nPac weekly and 72 with nPac d1,8 q22) and 64 deaths (28 with denosumab and 36 without denosumab; 29 with nPac weekly and 35 with nPac d1,8 q22) occurred in the total patient population. According to univariate analysis, there was no statistically significant difference in iDFS (HR 0.86, 95% CI 0.62-1.21, *P* = 0.39) (Figure 2), LRRFI (HR 1.41, 95% CI 0.76-2.63, *P* = 0.28), DDFS (HR 0.77, 95% CI 0.54-1.11, *P* = 0.16), and OS (HR 0.81, 95% CI 0.50-1.33, *P* = 0.41) (Figure 3) between the denosumab-treated and non-denosumab-treated arms.

In comprehensive subgroup analyses, none of the stratification factors, like breast cancer subtype, lymphocyte predominant breast cancer status, and EC schedule, or the breast cancer gene (*BRCA*) mutational status indicated a significant benefit from denosumab or one of the nPac regimens regarding IDFS, OS, and DDFS outcomes [Supplementary Figure S1, available at https://doi.org/10.1016/j.esmoop.2025.105915 (data shown only for DDFS and denosumab randomization)].

We subsequently analyzed the long-term effect of denosumab treatment on the above-mentioned outcome parameters using multivariate Cox regression analysis, including established breast cancer risk factors. Nodal status, tumor stage, and pCR status were confirmed as risk factors in all three multivariate models carried out (DDFS in Table 1, iDFS and OS in Supplementary Table S1, available at https://doi.org/10.1016/j.esmoop.2025.105915).

**Table 1:** Multivariate Cox regression analysis (landmark) for distant disease-free survival

Parameter	Category	Hazard ratio	95% CI	*P* value
Arm denosumab	With D	0.635	0.431-0.934	0.021
	Without D			
Arm chemotherapy	nPac weekly	0.871	0.597-1.271	0.475
	nPac d1,8			
Breast cancer subtype	HER2 negative/hormone receptor positive			0.115
	TNBC	1.518	0.973-2.366	0.066
	HER2 positive	0.921	0.502-1.691	0.791
LPBC	No LPBC (TILs < 50%)			
	LPBC (TILs > 50%)	0.328	0.103-1.048	0.060
Age, years	<40			
	40+	0.763	0.477-1.220	0.258
cN	cN0			
	cN+	2.140	1.439-3.183	<0.001
cT	cT1-3			
	cT4	4.993	2.110-11.81	<0.001
Grading	G1-G2			
	G3	1.325	0.872-2.013	0.188
EC schedule	2-weekly			
	3-weekly	1.158	0.789-1.702	0.453
Tumor size (mm)	≤25			
	>25	1.640	1.109-2.424	0.013
pCR (ypT0/is ypN0)	No			
	Yes	0.355	0.223-0.564	<0.001

c, clinical; CI, confidence interval; D, denosumab; d1,8, days 1 and 8; EC, epirubicin and cyclophosphamide; G, grade; HER2, human epidermal growth factor receptor 2; LPBC, lymphocyte predominant breast cancer; n, nodal; nPac, nab-paclitaxel; pCR, pathological complete response; T, tumor; TIL, tumor-infiltrating lymphocyte; TNBC, triple-negative breast cancer.

Overall, there were 90 distant relapses as the first event. A total of 36/90 distant relapses occurred in patients with denosumab treatment (9.2%), compared with 54/90 events in patients without denosumab (13.8%) (Supplementary Table S2, available at https://doi.org/10.1016/j.esmoop.2025.105915). Denosumab treatment resulted in a statistically significant risk reduction of 36% for DDFS in the multivariate (HR 0.64, 95% CI 0.43-0.93, *P* = 0.02; Table 1) but not in univariate analysis (HR 0.77, 95% CI 0.54-1.11, *P* = 0.16; Figure 3), although there was a trend in the subgroup of patients with pCR (HR 0.462, 95% CI 0.210-1.01, *P* = 0.054) (Supplementary Figure S2, available at https://doi.org/10.1016/j.esmoop.2025.105915). The multivariable analysis was conducted as a prespecified landmark Cox model with surgery as the time origin, allowing inclusion of pCR as a covariate alongside baseline clinicopathological factors. As a sensitivity analysis, we additionally carried out a baseline-only Cox model with randomization as the time origin and exclusion of pCR; this model showed an attenuated but directionally consistent, non-significant association (Supplementary Table S3, available at https://doi.org/10.1016/j.esmoop.2025.105915).

Subgroup-level data on DDFS showed no statistically significant difference in any individual breast cancer subtype, including HER2-positive, triple-negative, and hormone receptor-positive/HER2-negative disease (Supplementary Figure S1, available at https://doi.org/10.1016/j.esmoop.2025.105915), indicating that the observed effect of denosumab on DDFS in the overall cohort was not primarily driven by a specific subgroup.

Secondary malignancy was a rare event [5 (1.3%) with denosumab versus 11 (2.8%) without denosumab]. Overall, patients with a pCR had a significant better outcome with regard to iDFS (HR 0.42, 95% CI 0.29-0.61, *P* < 0.0001), DDFS (HR 0.35, 95% CI 0.23-0.53, *P* < 0.0001), and OS (HR 0.32, 95% CI 0.18-0.58, *P* = 0.0001) (Supplementary Figure S3, available at https://doi.org/10.1016/j.esmoop.2025.105915). This effect was particularly strong in patients with TNBC (iDFS; HR 0.22, 95% CI 0.12-0.39, *P* < 0.0001; Supplementary Figure S4C, available at https://doi.org/10.1016/j.esmoop.2025.105915). The presence of a TNBC subtype *per se* was an independent predictor of poor survival (HR 1.89, 95% CI 1.06-3.33, *P* = 0.03; Supplementary Table S1, available at https://doi.org/10.1016/j.esmoop.2025.105915). However, denosumab treatment did not improve iDFS in TNBC patients (HR 0.93, 95% CI 0.56-1.57, *P* = 0.8; Supplementary Figure S4A, available at https://doi.org/10.1016/j.esmoop.2025.105915).

Although nPac weekly resulted in a significantly higher pCR rate in TNBC,^23^ there was no overall differential impact of the two different NACT regimens (nPac weekly or nPac d1,8 q22) on long-term outcome in triple-negative patients (iDFS; HR 0.69, 95% CI 0.41-1.17, *P* = 0.17; Supplementary Figure S4B, available at https://doi.org/10.1016/j.esmoop.2025.105915).

Interestingly, TNBC patients with a non-pCR status showed a non-significant trend toward improved iDFS with the weekly nPac regimen, reflected in an absolute difference of 13.6% in 5-year iDFS rates (72.0% versus 58.4%; HR 0.58, 95% CI 0.29-1.15, *P* = 0.12; Supplementary Figure S4D, available at https://doi.org/10.1016/j.esmoop.2025.105915).

### Post hoc exploratory analysis of bone metastasis and RANK status

Considering the association between RANKL/RANK signaling and bone metastasis,^16^ we were interested in the long-term effect of neoadjuvant denosumab on bone metastases. Bone metastasis relapse rates (with bone metastasis as the first event) were 4.2% with denosumab and almost doubled without denosumab (7.6%) at the 5-year landmark (HR 0.59, 95% CI 0.31-1.12, *P* = 0.11; Supplementary Table S4A, available at https://doi.org/10.1016/j.esmoop.2025.105915). This effect was most pronounced in patients with hormone receptor-positive disease (3.9% with denosumab versus 8.0% without denosumab; HR 0.56, 95% CI 0.23-1.36, *P* = 0.17; Supplementary Table S4B, available at https://doi.org/10.1016/j.esmoop.2025.105915). However, none of these observations reached statistical significance.

As previously reported, a total of 667/780 pre-therapeutic core biopsies of the breast from the patients of the GeparX study were available and had been subjected to immunohistochemical detection of RANK expression.

RANK expression status did not show any impact on long-term iDFS, neither among denosumab-treated nor among non-denosumab-treated patients (HR 1.13, 95% CI 0.56-2.28, *P* = 0.74; Supplementary Figure S5, available at https://doi.org/10.1016/j.esmoop.2025.105915).

### Safety assessment

According to patient reports, symptoms suggestive of severe PNP occurred more frequently in the weekly nPac group (16.3%) compared with the d1,8 q22 group (7.8%) at any follow-up timepoint. However, this difference was not observed at the final assessment before data cut-off (7.5% nPac weekly versus 5.7% nPac d1,8 q22). It should be noted that PNP symptoms were assessed via patient self-reporting and do not reflect Common Terminology Criteria for Adverse Events (CTCAE) criteria (Supplementary Table S5, available at https://doi.org/10.1016/j.esmoop.2025.105915).

The total number of bone fractures (*n* = 45) was equally distributed between the denosumab (*n* = 23) and the non-denosumab (*n* = 22) arms (Supplementary Table S6, available at https://doi.org/10.1016/j.esmoop.2025.105915).

Within the on-treatment period, no atypical femoral fractures were reported. One clinically verified high-grade case of osteonecrosis of the jaw (ONJ) occurred in the denosumab group (0.3%; 0% in the control arm); these events were captured under protocol-defined, site-based safety surveillance.

## Discussion

In our present long-term study of GeparX, we report that the outcome parameters LRRFI, iDFS, DDFS, and OS were not significantly affected by denosumab add-on treatment in the univariate analysis. However, denosumab significantly reduced the incidence of distant metastasis in the multivariate analysis.

The fact that tumors show a predilection for metastasis to specific organ sites remains a well-established paradigm in cancer research. A growing body of experimental evidence has validated Stephen Pagets ‘seed and soil’ hypothesis, proposing that the metastatic preference of a tumor results from favorable interactions with specific organ microenvironments.^26^ As breast cancer is also an osteotropic tumor, bone metastasis constitutes a severe clinical complication in breast cancer patients, with particularly osteolytic bone metastases having a detrimental impact on quality of life and survival.^4^^,^^5^ Therefore, modifying the bone microenvironment by anti-resorptive agents, such as bisphosphonates or denosumab, is a reasonable clinical strategy, not only as prophylaxis of osteopenia and osteoporosis but also as antitumor treatment in patients with breast cancer. A meta-analysis by the Early Breast Cancer Trialists’ Collaborative Group (EBCTCG) showed that adjuvant bisphosphonate treatment reduced bone metastatic recurrences and improved breast cancer-specific survival in postmenopausal women, while additionally reducing fracture rates.^27^ For denosumab, clinical activity has already been shown in the adjuvant setting.^18^, ^19^, ^20^, ^21^, ^22^ The ABCSG-18 trial, for example, demonstrated that denosumab increased bone mineral density, delayed the time to first fracture, and improved DFS in postmenopausal women receiving adjuvant aromatase inhibitor therapy.^19^^,^^20^ In contrast, the D-CARE trial was negative with respect to survival outcomes.^22^ This discrepancy may be attributed to differences in patient populations, with ABCSG-18 including predominantly low-risk patients, while D-CARE enrolled a higher-risk cohort.

We report for the first time that denosumab could provide clinical benefit in the neoadjuvant setting with a relatively short course of denosumab for only 24 weeks by reducing the rate of distant relapses, further strengthening the concept that RANK signaling promotes metastatic breast cancer progression.^14^, ^15^, ^16^ However, we did not observe a benefit among the other outcome parameters LRRFI, iDFS, and OS.

Our analysis was planned to address prognosis conditional on post-treatment response. A landmark framework with surgery as the time origin permits inclusion of pCR, the most established short-term surrogate in the neoadjuvant setting, and answers a different clinical question than a baseline-only model that ignores response. The baseline-only sensitivity analysis produced a concordant but attenuated and non-significant association, which supports cautious interpretation of the cohort-level signal and underscores the clinical relevance of conditioning on pCR.

Our results are not directly comparable to previous trials on denosumab, as those have been carried out in a different clinical setting. In the ABCSG-18 trial, denosumab was administered, according to its approval for the treatment of osteoporosis (60 mg every 6 months with a median number of seven doses), whereas the dosage used in GeparX was much higher (approved dosage for patients with bone metastases) with a noticeably shorter treatment duration (120 mg every 4 weeks for six cycles). All patients from the ABCSG-18 trial were hormone receptor positive/HER2 negative, while this subtype was represented by only 40% in the GeparX trial.^23^ Moreover, all GeparX patients were subjected to NACT, due to the high-risk nature of the enrolled breast cancer subtypes, whereas in the ABCSG-18 trial, only 5% and 20% received neoadjuvant or adjuvant chemotherapy, respectively. Additionally, in the GeparX study, median patient age was notably younger with 49 compared with 64 years in ABCSG-18, which has an impact on risk and bone metabolism as well as fracture rates.^20^^,^^23^ As the majority of ABCSG-18 patients were subjected to aromatase inhibition as the only systemic treatment, stimulation of bone metabolism by the opposing interaction of endocrine therapy versus anti-resorptive denosumab could additionally improve patients’ outcome.

Despite some numerical trends, neoadjuvant denosumab treatment in the GeparX trial did not show a statistically significant effect on outcome parameters other than DDFS. One possible explanation is the high proportion of hormone receptor-positive/HER2-negative patients (39.7%), a subgroup that typically has long intervals between initial diagnosis and recurrence.^28^ For this subtype, the median follow-up of 62.3 months may have been too short to reveal differences in long-term outcomes. Moreover, DDFS is a clinically meaningful endpoint, as patients experiencing a DDFS event are likely to die of breast cancer with longer follow-up. Therefore, DDFS serves as an early but highly relevant indicator of long-term prognosis and might represent a surrogate endpoint for OS—particularly in studies with limited follow-up duration. OS of metastatic hormone receptor-positive/HER2-negative patients has continuously been improved over the last years, due to the development of next-generation ER-targeted therapies^29^ or novel antibody–drug conjugates.^30^ We suppose that a longer follow-up of 10-15 years, providing higher temporal resolution of events in hormone receptor-positive/HER2-negative patients, particularly after discontinuation of endocrine therapy, could unmask broader clinical benefits of neoadjuvant denosumab on the patient’s outcome. Additionally, the estimated 5-year cumulative incidence rate for distant diseases/death in HER2-positive and TNBC patients was relatively low (12.3% HER2 positive and 17.3% TNBC). Beyond that, we would not expect a denosumab effect on local recurrences.

In the GeparSepto trial, pCR rates and iDFS were significantly improved by the use of nPac weekly compared with solvent-based paclitaxel, yet no improvement in OS has been shown.^8^^,^^9^ These results provided rationale for nPac as the taxane of choice in the GeparX trial. Due to the higher rates of high-grade peripheral sensory neuropathy with nPac, the second randomization in GeparX was used to investigate whether a break in the nPac regimen would result in comparable outcomes (weekly versus d1,8 q22).^23^ Despite an increase in pCR by weekly nPac, we did not observe a differential effect of this regimen on the long-term outcome in the GeparX patients. Here again, a longer follow-up with more events in hormone receptor-positive/HER2-negative patients should be considered for a final conclusion. Notably, TNBC patients under the nPac weekly regimen showed a trend for a better iDFS in the landmark analysis at year 5, particularly those without pCR. Considering the risks and benefits, we suggest that the weekly nPac regimen could be the preferred treatment for TNBC patients only.

Several limitations of the present long-term follow-up analysis must be taken into account when interpreting the safety and outcome data. Firstly, while no unexpected toxicities were observed, the safety assessment is limited by missing data and the absence of standardized toxicity grading according to the CTCAE, as data are based primarily on patient-reported questionnaires. As a result, findings—particularly regarding PNP and other long-term toxicities—should be interpreted with caution. Secondly, although no difference in residual PNP was observed between the two nPac regimens, and fracture rates were comparable between denosumab and control arms, the available data did not allow differentiation between traumatic and osteoporotic fractures. In addition, information on the use of adjuvant bisphosphonates or denosumab after the neoadjuvant phase was not available. While such treatments were permitted according to clinical guidelines, their use was not documented in the study protocol. As care beyond surgery followed routine standards and allocation was randomized, systematic differences between groups are unlikely. If both arms received anti-resorptive agents to a similar extent, any resulting effect would more likely have attenuated than inflated the observed association. The DDFS findings should therefore be interpreted with appropriate caution.

While patient-reported follow-up is limited in accuracy, skeletal safety was prospectively monitored during treatment, with no atypical femoral fractures observed and only one clinically verified case of ONJ in the denosumab group. Given the short exposure period (six 120 mg doses over 24 weeks) and the pharmacologic properties of denosumab, which is not retained in the bone matrix, long-term skeletal hazard attributable to the study drug appears biologically unlikely.

Despite a reported superior efficacy in neoadjuvant treatment,^8^ the use of nPac, which is only approved in the metastatic setting, may limit the generalizability of results. Furthermore, the treatment landscape in TNBC has changed significantly since the trial was conducted, with the integration of immune checkpoint inhibitors such as pembrolizumab into the neoadjuvant setting leading to improved event-free survival and OS.^31^ In light of these developments, the current findings are not directly transferable to today’s TNBC standard of care. Lastly, the low event rate in HER2-positive patients restricts the ability to draw conclusions for this subgroup. These limitations highlight the need for cautious interpretation of the long-term results within the evolving clinical context. Nevertheless, the data provide important insights into long-term safety and outcomes, particularly in hormone receptor-positive patients, and may serve as a valuable reference for future comparative studies.

### Conclusion

GeparX is the first study worldwide investigating neoadjuvant denosumab treatment in high-risk early breast cancer patients. In the present 5-year follow-up, denosumab was associated with a reduction in distant metastases, without a corresponding improvement in OS. Similarly, the weekly nPac regimen did not result in long-term outcome improvement, despite previously reported pCR advantages. These findings provide important clinical insight by clarifying that intensified neoadjuvant strategies may improve distant disease control without necessarily translating into survival benefit within the first 5 years. Further follow-up is warranted, in order to potentially unmask broader clinical benefits of neoadjuvant treatment on the patient’s outcome, especially for the hormone receptor-positive/HER2-negative subtype. In the interim, this dataset provides relevant evidence to guide treatment decisions and inform the design of future neoadjuvant trials in high-risk early breast cancer.
