# Supplementary material for: Long-term effect of neoadjuvant denosumab treatment in high-risk early breast cancer (GeparX)
Source: ESMO Open. 2025 Nov 27;10(12):105915. doi: 10.1016/j.esmoop.2025.105915 (PMC12702335; doi:10.1016/j.esmoop.2025.105915)
Supplement: Supplemental Material [file mmc1.pdf]

## SUPPLEMENTAL MATERIAL

**Supplementary Figure 1**: Forest plots for DDFS in subgroups (denosumab randomization), Cox regression

| Subgroup | N patients | Hazard Ratio (95% CI) | p-Value | Test for Interaction |
|---|---|---|---|---|
| **Overall** | 780 | .770 (.535, 1.11) | 0.157 | |
| **subtype** | | | | 0.895 |
| HER2-/HR+ | 310 | .845 (.494, 1.45) | 0.539 | |
| TNBC | 317 | .744 (.424, 1.31) | 0.303 | |
| HER2+ | 153 | .647 (.230, 1.82) | 0.409 | |
| **LPBC** | | | | 0.358 |
| no LPBC (TILs<50%) | 718 | .742 (.514, 1.07) | 0.113 | |
| LPBC (TILs>50%) | 62 | 2.34 (.212, 25.8) | 0.487 | |
| **EC schedule** | | | | 0.608 |
| 2-weekly | 414 | .703 (.426, 1.16) | 0.168 | |
| 3-weekly | 366 | .850 (.502, 1.44) | 0.545 | |
| **Path mut in BRCA1 and BRCA2 only** | | | | 0.639 |
| wt | 676 | .784 (.535, 1.15) | 0.213 | |
| mt | 91 | .585 (.171, 2.00) | 0.393 | |
| **Path mut in BRCA1 and BRCA2 and 16 further genes** | | | | 0.213 |
| wt | 617 | .848 (.570, 1.26) | 0.418 | |
| mt | 150 | .435 (.167, 1.13) | 0.088 | |
| **Mut in 16 non BRCA1/2 genes vs no mut** | | | | 0.205 |
| wt | 617 | .848 (.570, 1.26) | 0.418 | |
| mt | 59 | .287 (.060, 1.38) | 0.120 | |

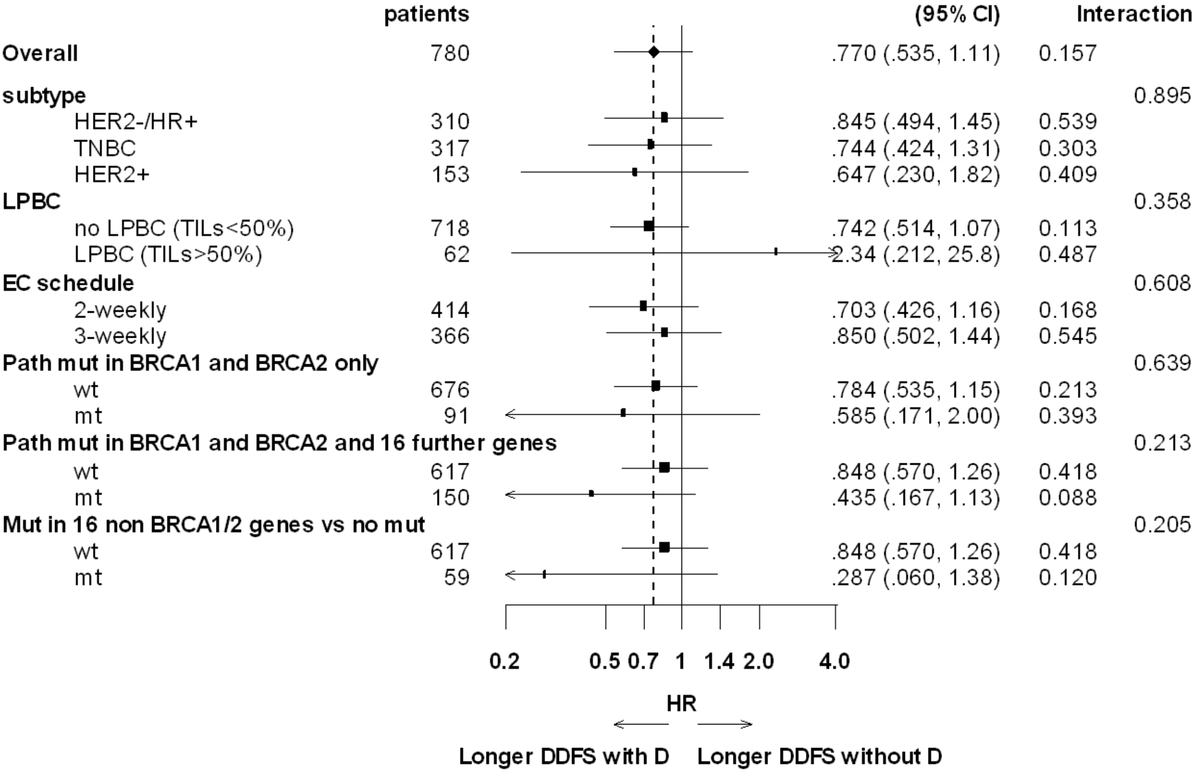


BRCA1/2 breast cancer gene 1/2; CI confidence interval; D denosumab; EC epirubicin and cyclophosphamide; HER2 human epithelial growth factor receptor 2; HR hormone receptor; iDFS invasive disease-free survival; LPBC lymphocyte predominant breast cancer; mt mutated; mut mutation; TILs tumor-infiltrating lymphocytes; TNBC triple negative breast cancer; wt wildtype; n.a. not available.

**Supplementary Figure 2:** Kaplan-Meier curves for distant disease-free survival according to denosumab treatment stratified for pCR

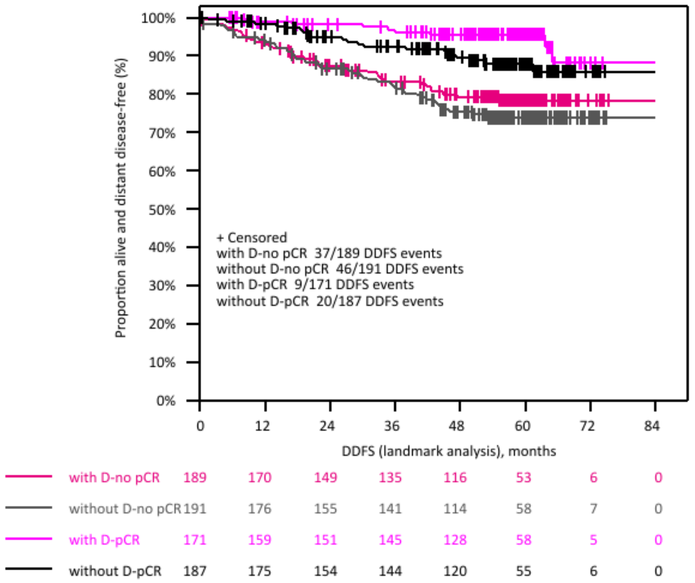


| Factor | subgroup | N | N events | HR D : without D | 95% CI for HR | p-value | p-value of interaction test |
|---|---|---|---|---|---|---|---|
| pCR (ypT0/is ypN0) | no | 380 | 83 | .821 | (0.532, 1.27) | 0.372 | 0.216 |
| | yes | 358 | 29 | .462 | (0.210, 1.01) | 0.054 | |

**Supplementary Figure 3**: Kaplan-Meier curves (landmark) for A) invasive disease-free survival, B) overall survival and C) distant disease-free survival, stratified for pCR (pCR blue, non-pCR black).

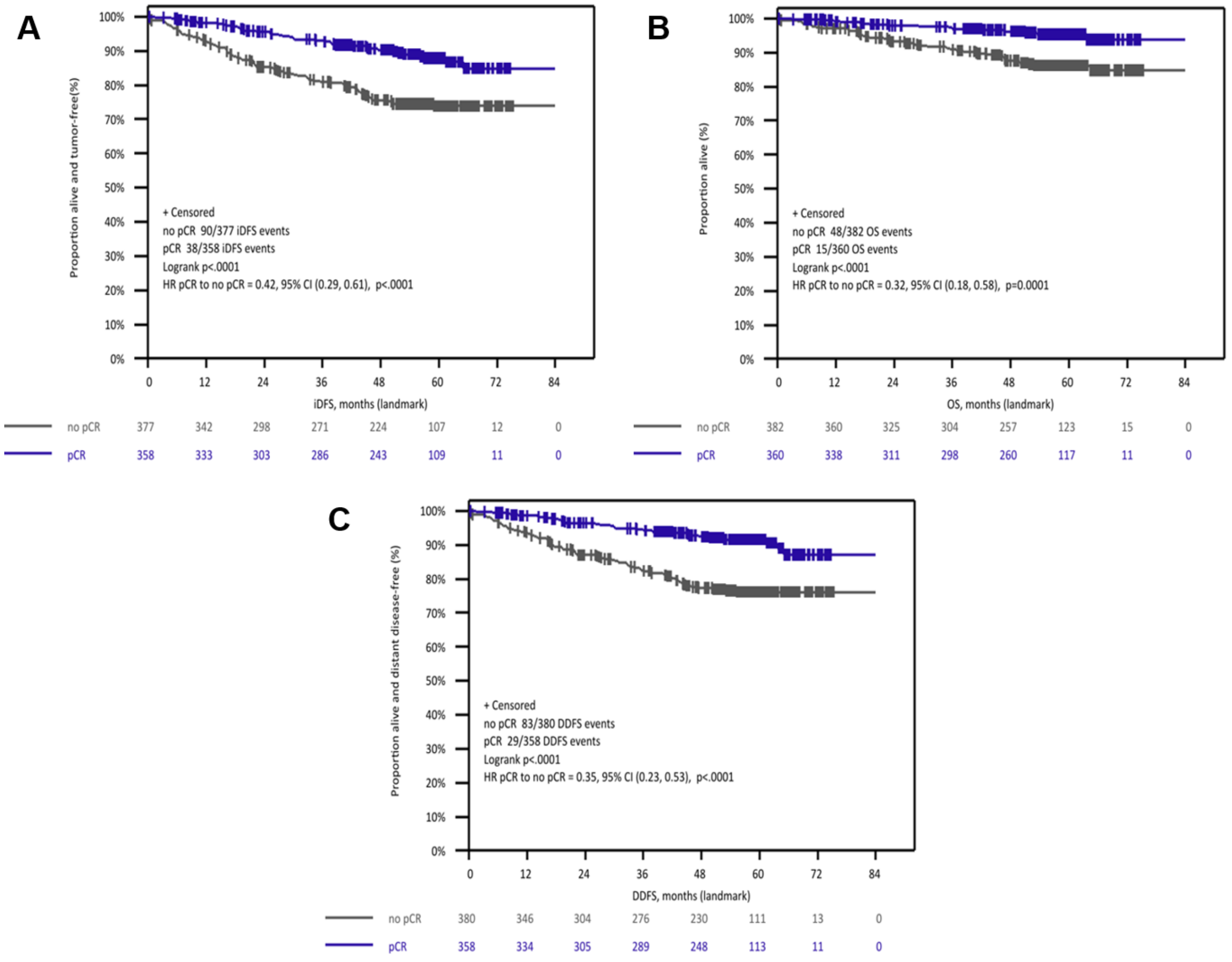


CI confidence interval; DDFS distant disease-free survival; HR hazard ratio; iDFS invasive disease-free survival; pCR pathological complete response; OS overall survival

**Supplementary Figure 4:** Kaplan-Meier curves in **TNBC** patients only for invasive disease-free survival for A) denosumab randomization B) nab-Paclitaxel randomization, C) pathological complete response (landmark analysis) and D) pathological complete response and nab-Paclitaxel randomization (landmark analysis)

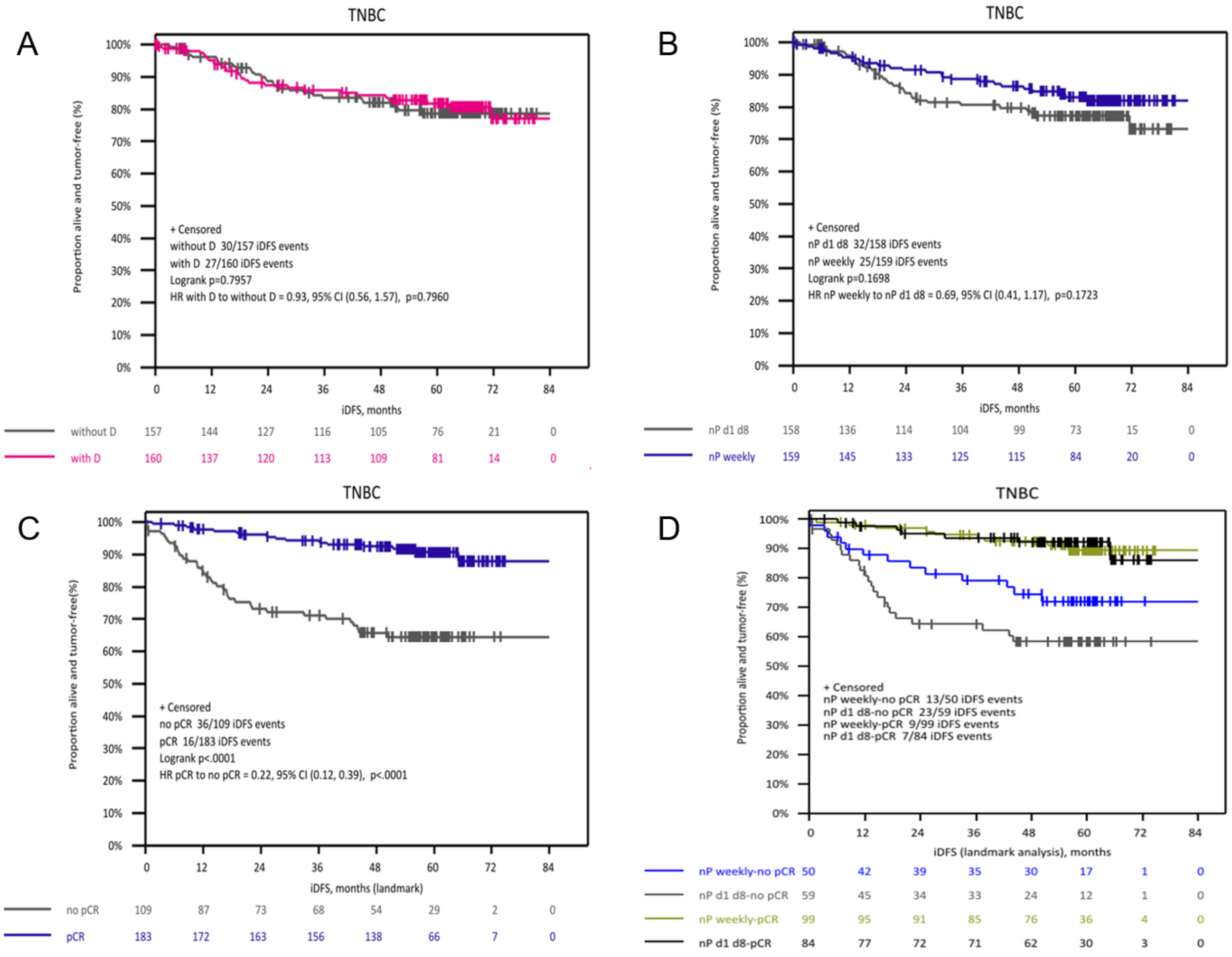


CI confidence interval; D denosumab; HR hazard ratio; iDFS invasive disease free survival; nP nab-Paclitaxel; pCR pathological complete response; TNBC triple negative breast cancer

**Supplementary Figure 5**: Kaplan-Meier curves for invasive disease-free survival stratified for **RANK**

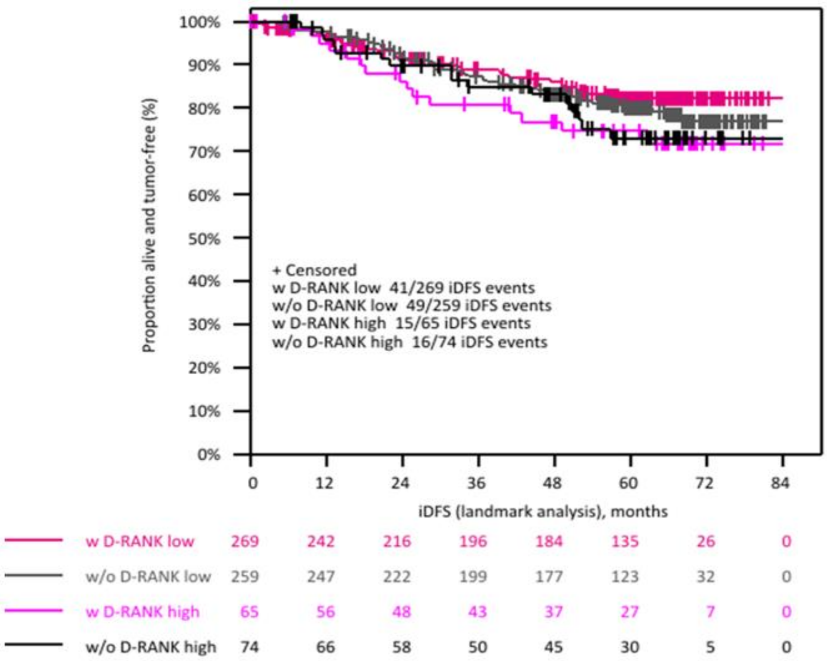


| IDFS analysis of RANK, dichotomized at Q3, denosumab arm | | | | | | | |
|---|---|---|---|---|---|---|---|
| **Factor** | **subgroup** | **N** | **N events** | **HR D: without D** | **95% CI for HR** | **p-value** | **p-value of interaction test** |
| Overall | Overall | 780 | 138 | .863 | (.618, 1.21) | 0.391 | |
| RANK, % stained | low | 528 | 90 | .840 | (.555, 1.27) | 0.410 | 0.491 |
| | high | 139 | 31 | 1.13 | (.557, 2.28) | 0.741 | |

CI confidence interval; D denosumab; HR hazard ratio; iDFS invasive disease-free survival; RANK Receptor activator of nuclear factor-kappa B; w with; w/o without

**Supplementary Table 1:** Multivariate Cox regression analysis (landmark) for invasive disease-free survival and overall survival.

| Parameter | Category | Hazard Ratio | IDFS 95% CI | IDFS p value | Hazard Ratio | OS 95% CI | OS p value |
|---|---|---|---|---|---|---|---|
| Arm denosumab | With D | 0.727 | (0.510, 1.038) | 0.080 | 0.679 | (0.405, 1.139) | 0.143 |
| | Without D | | | | | | |
| Arm chemotherapy | nP weekly | 0.907 | (0.638, 1.289) | 0.585 | 0.896 | (0.539, 1.490) | 0.672 |
| | nP d1 d8 | | | | | | |
| Breast cancer subtype | HER2-/HR+ | | | 0.211 | | | 0.015 |
| | TNBC | 1.416 | (0.929, 2.160) | 0.106 | 1.876 | (1.057, 3.327) | 0.032 |
| | HER2+ | 0.981 | (0.569, 1.691) | 0.945 | 0.503 | (0.173, 1.463) | 0.207 |
| LPBC | no LPBC (TILs<50%) | | | | | | |
| | LPBC (TILs>50%) | 0.365 | (0.133, 1.002) | 0.050 | 0.176 | (0.024, 1.290) | 0.087 |
| Age | <40 | | | | | | |
| | 40+ | 0.749 | (0.485, 1.156) | 0.191 | 0.899 | (0.461, 1.751) | 0.754 |
| cN | cN0 | | | | | | |
| | cN+ | 2.116 | (1.460, 3.066) | <0.001 | 2.688 | (1.557, 4.643) | <0.001 |
| cT | cT1-3 | | | | | | |
| | cT4 | 4.186 | (1.789, 9.796) | <0.001 | 5.843 | (2.215, 15.42) | <0.001 |
| Grading | G1-G2 | | | | | | |
| | G3 | 1.381 | (0.931, 2.049) | 0.108 | 1.474 | (0.830, 2.616) | 0.185 |
| EC schedule | 2-weekly | | | | | | |
| | 3-weekly | 1.111 | (0.776, 1.590) | 0.565 | 1.318 | (0.784, 2.216) | 0.297 |
| Tumor size (mm) | <=25 | | | | | | |
| | >25 | 1.578 | (1.097, 2.270) | 0.014 | 1.434 | (0.851, 2.416) | 0.176 |
| pCR (ypT0/is ypN0) | no | | | | | | |
| | yes | 0.421 | (0.276, 0.640) | <0.001 | 0.326 | (0.172, 0.616) | <0.001 |

C clinical; CI confidence interval; D denosumab; EC epirubicin and cyclophosphamide; G grading; HER2 human epithelial growth factor receptor 2; HR hormone receptor; iDFS invasive disease-free survival; LPBC lymphocyte predominant breast cancer; n nodal; nP nab-Paclitaxel; OS overall survival; pCR pathological complete response; T tumor; TILs tumor-infiltrating lymphocytes; TNBC triple negative breast cancer.

**Supplementary Table 2:** First distant disease-free survival event, secondary malignancy and death accordingly to denosumab rando

| | With D | Without D | Overall |
|---|---|---|---|
| **Distant relapse** | 36 (9.2) | 54 (13.8) | 90 (11.5) |
| **Secondary malignancy** | 5 (1.3) | 11 (2.8) | 16 (2.1) |
| **Death** | 10 (2.6) | 3 (0.8) | 13 (1.7) |

D denosumab

**Supplementary Table 3:** Multivariate Cox regression analysis for distant disease-free survival without pCR.

| | | **Multivariate model without pCR** | | |
|---|---|---|---|---|
| **Parameter** | **Category** | **Hazard Ratio** | **95% CI** | **p value** |
| Arm denosumab | With D | 0.698 | (0.483, 1.010) | 0.057 |
| | Without D | | | |
| Arm chemotherapy | nP weekly | 0.844 | (0.586, 1.214) | 0.360 |
| | nP d1 d8 | | | |
| Breast cancer subtype | HER2-/HR+ | | | .282 |
| | TNBC | 1.091 | (0.719, 1.656) | 0.683 |
| | HER2+ | 0.677 | (0.380, 1.206) | 0.185 |
| LPBC | no LPBC (TILs<50%) | | | |
| | LPBC (TILs>50%) | 0.275 | (0.087, 0.873) | 0.029 |
| Age | <40 | | | |
| | 40+ | 0.821 | (0.524, 1.284) | 0.387 |
| cN | cN0 | | | |
| | cN+ | 2.106 | (1.439, 3.082) | <0.001 |
| cT | cT1-3 | | | |

| | | | | |
|---|---|---|---|---|
| | cT4 | 5.351 | (2.434, 11.76) | <0.001 |
| Grading | G1-G2 | | | |
| | G3 | 1.167 | (0.778, 1.751) | 0.456 |
| EC schedule | 2-weekly | | | |
| | 3-weekly | 1.163 | (0.804, 1.682) | 0.424 |
| Tumor size (mm) | <=25 | | | |
| | >25 | 1,798 | (1.237, 2.613) | 0.002 |

C clinical; CI confidence interval; D denosumab; EC epirubicin and cyclophosphamide; G grading; HER2 human epithelial growth factor receptor 2; HR hormone receptor; LPBC lymphocyte predominant breast cancer; n nodal; nP nab-Paclitaxel; pCR pathological complete response; T tumor; TILs tumor-infiltrating lymphocytes; TNBC triple negative breast cancer.

**Supplementary Table 4:** Bone metastasis relapse rates (bone metastasis as first event, other simultaneous events ignored), denosumab randomization A) ITT; B) only hormone receptor positive patients.

A

| | **With D** | **Without D** |
|---|---|---|
| **Estimated 3 year BMR CIR, %** | 2.9% (1.5%, 5.0%) | 3.8% (2.2%, 6.2%) |
| **Estimated 4 year BMR CIR, %** | 3.5% (1.9%, 5.8%) | 5.4% (3.3%, 8.1%) |
| **Estimated 5 year BMR CIR, %** | 4.2% (2.4%, 6.7%) | 7.6% (5.0%, 10.8%) |
| **HR (95% CI)** | 0.592 (0.313, 1.120) | |
| **Gray p-value** | 0.1075 | |
| **(Stratified) Gray p-value** | 0.1068 | |

B

| | **With D** | **Without D** |
|---|---|---|
| **Estimated 3 year BMR CIR, %** | 2.7% (1.0%, 5.8%) | 3.6% (1.6%, 6.9%) |
| **Estimated 4 year BMR CIR, %** | 3.3% (1.3%, 6.6%) | 5.8% (3.1%, 9.8%) |
| **Estimated 5 year BMR CIR, %** | 3.9% (1.7%, 7.6%) | 8.0% (4.5%, 12.6%) |
| **HR (95% CI)** | 0.563 (0.234, 1.355) | |
| **Gray p-value** | 0.1741 | |

| (Stratified) Gray p-value | 0.1999 | |
|---|---|---|

BMR bone metastasis relapse; CI confidence interval; CIR cumulative incidence rate; D denosumab; HR hazard ratio

## **Supplementary Table 5:** Peripheral sensory neuropathy rates from patients self reported data

| Parameter | Assessment by PSR | nP weekly, n= 258 (100%) | nP d1 d8, n= 271 (100%) | Overall, n= 529 (100%) |
|---|---|---|---|---|
| **Peripheral sensory neuropathy (PNP)*, max. over all evaluations** | unknown or AESI not present | 79 (30.7) | 134 (49.6) | 213 (40.4) |
| | mild or moderate | 136 (52.9) | 113 (41.9) | 249 (47.2) |
| | severe | 42 (16.3) | 21 (7.8) | 63 (12.0) |
| | severity unknown | 0 (0.0) | 2 (0.7) | 2 (0.4) |
| | missing | 1 | 1 | 2 |
| **PNP assessment at last evaluation** | unknown or AESI not present | 114 (45.2) | 162 (62.1) | 276 (53.8) |
| | mild or moderate | 119 (47.2) | 83 (31.8) | 202 (39.4) |
| | severe | 19 (7.5) | 15 (5.7) | 34 (6.6) |
| | severity unknown | 0 (0.0) | 1 (0.4) | 1 (0.2) |
| | missing | 6 | 10 | 16 |

AESI adverse event of special interest; nP nab-paclitaxel; PSR patient self reported data

## **Supplementary Table 6:** Fracture rates with and without Denosumab treatment

| Parameter | Subjective assessment or assessment by centre | With D, n= 320 (100%) | Without D, n= 330 (100%) | Overall, n= 650 (100%) |
|---|---|---|---|---|
| **Patients with at least one bone fracture** | unknown or AESI not present | 250 (91.6) | 253 (92.0) | 503 (91.8) |
| | mild or moderate | 11 (4.0) | 12 (4.4) | 23 (4.2) |
| | severe | 6 (2.2) | 5 (1.8) | 11 (2.0) |
| | severity unknown | 6 (2.2) | 5 (1.8) | 11 (2.0) |
| | missing | 47 | 55 | 102 |

AESI adverse event of special interest; D denosumab
